# Immune‐Related Adverse Events in Bladder Cancer Patients Treated With Immune Checkpoint Inhibitors: Insights From a FAERS Disproportionality Analysis

**DOI:** 10.1002/cam4.71777

**Published:** 2026-04-02

**Authors:** Pooja Gokhale, Lorenzo Villa Zapata

**Affiliations:** ^1^ Department of Clinical and Administrative Pharmacy University of Georgia College of Pharmacy Athens Georgia USA

**Keywords:** bladder cancer, disproportionality analysis, FDA adverse event reporting system (FAERS), immune checkpoint inhibitors, immune‐related adverse events

## Abstract

**Introduction:**

Immune checkpoint inhibitors (ICIs) are an important treatment option in bladder cancer, but clinical trials have demonstrated that they are associated with immune‐related adverse events (irAEs), such as rash, hypothyroidism, hyperthyroidism, and others. This study aimed to evaluate real‐world evidence of these irAEs using the FDA Adverse Event Reporting System (FAERS).

**Methods:**

We analyzed FAERS data through December 2024 to identify irAEs associated with atezolizumab, pembrolizumab, avelumab, and nivolumab in patients with bladder cancer. A case/non‐case pharmacovigilance analysis was conducted to assess the association between ICI monotherapy and specific irAEs, including rash, pruritus, hypothyroidism, hyperthyroidism, colitis, nephritis, myocarditis, pneumonitis, and myositis.

**Results:**

A total of 365 unique irAE reports were identified. Significant safety signals were observed for hyperthyroidism with atezolizumab (PRR: 3.51 [95% CI: 1.07–11.45]; ROR: 3.53 [95% CI: 1.07–11.61]; IC: 1.10 [95% CI: 0.33–1.87]), rash with pembrolizumab (PRR: 2.11 [95% CI: 1.17–3.80]; ROR: 2.14 [95% CI: 1.18–3.90]; IC: 0.44 [95% CI: 0.17–0.71]), and myositis with avelumab (PRR: 5.30 [95% CI: 1.35–20.82]; ROR: 5.47 [95% CI: 1.34–22.29]; IC: 1.95 [95% CI: 0.65–3.25]). While not reaching the threshold for statistical significance, nephritis with atezolizumab did not meet predefined signal detection criteria and should only be considered as hypothesis‐generating. A similar borderline pattern was observed for hypothyroidism with avelumab.

**Conclusion:**

The occurrence of irAEs varies by ICI agent among bladder cancer patients. These findings underscore the importance of post‐marketing surveillance in identifying real‐world safety signals. Proactive management and patient education are essential to mitigate irAEs and preserve quality of life.

## 1 Introduction

Bladder cancer is a malignancy that primarily affects the urinary system and ranks as the ninth most common cancer worldwide [1]. In the United States (US), approximately 84,870 new cases of bladder cancer were reported in 2024, according to the American Cancer Society [2]. The most prevalent form is urothelial carcinoma, which accounts for about 90% of all cases [3].

Urothelial carcinoma can be categorized into two major types based on the nature of the lesion. Non‐muscle invasive bladder cancer (NMIBC) is characterized by low‐grade papillary tumors, and muscle‐invasive bladder cancer (MIBC), which typically arises from flat dysplasia or carcinoma in situ [3]. Treatment strategies vary depending on disease stage and may include surgical resection, intravesical therapy, Bacillus–Calmette–Guérin (BCG) therapy, radiotherapy, and immunotherapy [3, 4]. Among these, immune checkpoint inhibitors (ICIs) have emerged as a key component in the therapeutic arsenal for bladder cancer [5].

The National Comprehensive Cancer Network (NCCN) guidelines recommend four ICIs as adjuvant, first, or second‐line systemic treatment: Atezolizumab, pembrolizumab, nivolumab, and avelumab [6]. ICIs fall into three primary categories: Cytotoxic T‐lymphocyte–associated protein 4 (CTLA‐4) inhibitors, programmed death‐1 (PD‐1) inhibitors, and programmed death‐ligand 1 (PD‐L1) inhibitors [7]. CTLA‐4 acts primarily within lymphoid tissues, while PD‐1 and PD‐L1 function in peripheral tissues to downregulate immune activity [8]. By inhibiting these regulatory pathways, ICIs promote T‐cell activation and enhance antitumor response [7].

However, while ICIs demonstrate significant antitumor efficacy, their immune‐modulating effects can also trigger immune‐related adverse events (irAEs). These include endocrinopathies (e.g., hypothyroidism, hyperthyroidism), gastrointestinal toxicities (e.g., colitis), and dermatologic or pulmonary reactions, among others. Notably, CTLA‐4 inhibitors have been associated with more severe irAEs compared to PD‐1 or PD‐L1 inhibitors [9]. Of the ICIs approved for bladder cancer, pembrolizumab and nivolumab are PD‐1 inhibitors, while avelumab and atezolizumab are PD‐L1 inhibitors [10].

Despite increasing use of ICIs in bladder cancer, limited real‐world evidence exists comparing their safety profiles. Although a systematic review reported irAEs in approximately 40% of patients treated with ICIs across various cancer types [11], comparative post‐marketing data for individual ICIs in bladder cancer remain scarce. No prior study has systematically evaluated the differential reporting of irAEs among these four ICIs using real‐world data from the FDA Adverse Event Reporting System (FAERS).

Spontaneous reporting systems such as FAERS provide a valuable source of real‐world pharmacovigilance data. When analyzed using disproportionality methods, these databases can help identify safety signals by detecting whether specific drug–event combinations are reported more frequently than expected relative to a background population [12].

This study aims to evaluate and compare the reporting rates of immune‐related adverse events associated with atezolizumab, avelumab, pembrolizumab, and nivolumab in patients with bladder cancer using data from the FAERS database.

## 2 Methods

This study adheres to the REporting of A Disproportionality Analysis for DrUg Safety Signal Detection Using Individual Case Safety Reports in PharmacoVigilance (READUS‐PV) guidelines [13].

### 2.1 Study Design and Data Source

We conducted a retrospective case/non‐case pharmacovigilance study to assess the association between ICIs and immune‐related adverse events irAEs in patients with bladder cancer using data from the FDA Adverse Event Reporting System (FAERS). FAERS is a spontaneous reporting database maintained by the U.S. Food and Drug Administration (FDA) for post‐marketing safety surveillance. It includes voluntary reports of adverse drug events submitted by healthcare professionals, pharmaceutical manufacturers, and consumers.

### 2.2 Data Collection

**FIGURE 1:**
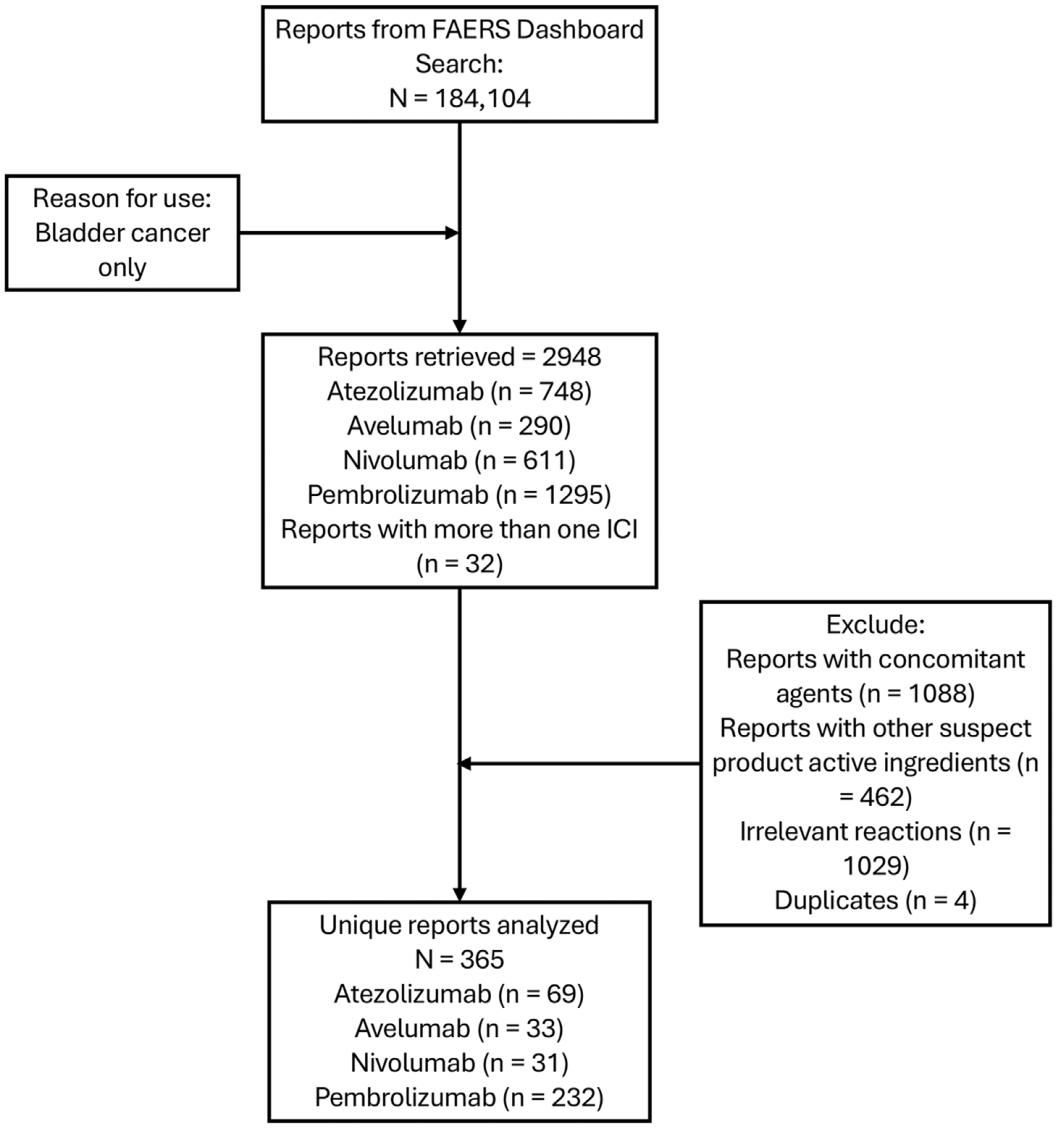
Flowchart of data collection.

We queried the FAERS database using the generic names of four ICIs: Atezolizumab, avelumab, nivolumab, and pembrolizumab. The indication was restricted to bladder cancer of any type or stage. We downloaded reports available through December 2024 from the FAERS dashboard. Reports were excluded if they contained irrelevant reactions, additional suspect drug ingredients, or concomitant medications likely to confound attribution. Duplicate records, defined as those with matching age, weight, FDA received date, country of occurrence, suspected active ingredient, and reported reaction, were also removed. The complete data selection process is shown in Figure 1.

### 2.3 Outcomes

Adverse events were classified using Preferred Terms (PTs) from the Medical Dictionary for Regulatory Activities (MedDRA version 28.0). Given the lack of a universally accepted definition for irAEs, we grouped events using MedDRA System Organ Classes (SOCs), following the approach described by Chen et al. (2021) [14]. These included: Skin and subcutaneous tissue disorders, gastrointestinal disorders, nervous system disorders, endocrine disorders, cardiac disorders, respiratory and thoracic mediastinal disorders, renal and urinary disorders, musculoskeletal and connective tissues disorders, and eye disorders. Adverse events that were infrequently reported were grouped under the category “other”. The details of the reported terms under each group are provided in Table S1. Our analysis focused on irAEs commonly observed in clinical trials [15, 16, 17, 18, 19]. These included: Hypothyroidism, hyperthyroidism, rash, pruritus, colitis, hepatitis, nephritis, myocarditis, pneumonitis, and myositis.

### 2.4 Statistical Analysis

Descriptive statistics, including frequencies and percentages, were calculated to summarize the characteristics of immune‐related adverse event reports by ICI type. Variables extracted from the reports included patient age, sex, reaction severity, clinical outcomes, reporter type, and country of origin. To detect safety signals, disproportionality analysis was conducted using three standard pharmacovigilance metrics: The reporting odds ratio (ROR), proportional reporting ratio (PRR), and information component (IC). Each measure was calculated along with its 95% confidence intervals (CIs). A signal was defined as statistically significant when the number of reported events was three or more, the lower limit of the 95% CI for both the ROR and PRR was greater than or equal to one, and the lower bound of the IC, referred to as IC_025_, was greater than or equal to zero. These criteria are commonly used to detect potential safety signals that may indicate a causal association between a drug and an adverse event and warrant further investigation (Bate & Evans, 2009) [20], [21] Each ICI was compared against all other ICIs used for bladder cancer to ensure that results reflect differences within the relevant clinical context, rather than all drugs across the FAERS database.

## 3 Results

### 3.1 Report Characteristics

A total of 2948 adverse event reports related to the use of atezolizumab, pembrolizumab, nivolumab, and avelumab in bladder cancer were identified in the FAERS database. After applying exclusion criteria and de‐duplication procedures, 365 reports were included in the final analysis (Figure 1). The majority of reports were associated with pembrolizumab (*n* = 232, 63.6%), followed by atezolizumab (*n* = 69, 18.9%), avelumab (*n* = 33, 9.0%), and nivolumab (*n* = 31, 8.5%). As shown in Table 1, over 80% of the reports were submitted by healthcare professionals, and approximately 43% originated from the United States. The mean age of patients in reports involving avelumab was higher (approximately 72 years) compared to those involving other ICIs, which had a mean age of around 65 years. Across all ICI types, adverse event reports were more commonly submitted for male patients. The majority of reported reactions were classified as serious, with over 80% of cases meeting this criterion and up to 90% of avelumab‐associated events reported as serious. Hospitalization rates varied by ICI, ranging from 22.6% for nivolumab to 40.6% for atezolizumab.

**TABLE 1:** Characteristics of the reports.

Attribute	Atezolizumab (*n* = 69)	Avelumab (*n* = 33)	Nivolumab (*n* = 31)	Pembrolizumab (*n* = 232)
Age, mean years ± SD	64.5 ± 13.2	72.2 ± 7.3	68.5 ± 13.2	66.6 ± 19.7
Sex, *n* (%)				
Male	41 (59.4)	25 (75.8)	18 (58.1)	175 (75.4)
Female	16 (23.2)	6 (18.2)	5 (16.1)	49 (21.1)
Not Specified (Sex)	12 (17.4)	2 (6.0)	8 (25.8)	8 (3.5)
**Severity of reaction, *n* (%)**				
Serious	57 (82.6)	30 (90.9)	25 (80.6)	193 (83.2)
Non‐Serious (Reaction)	12 (17.4)	3 (9.1)	6 (19.4)	39 (16.8)
**Outcomes, *n* (%)**				
Died	5 (7.2)	5 (15.2)	2 (6.5)	30 (12.9)
Hospitalized	28 (40.6)	9 (27.3)	7 (22.6)	78 (33.6)
Disabled	1 (1.4)	0 (0.0)	0 (0.0)	2 (0.8)
Life Threatening	0 (0.0)	1 (3.0)	0 (0.0)	7 (3.0)
Non‐Serious (Outcome)	12 (17.4)	3 (9.1)	6 (19.3)	39 (16.8)
Other Outcomes	23 (33.4)	15 (45.4)	16 (51.6)	76 (32.9)
**Reporter type, *n* (%)**				
Healthcare Professional	64 (92.8)	31 (93.9)	25 (80.6)	194 (83.6)
Consumer	5 (7.2)	2 (6.1)	6 (19.4)	38 (16.4)
**Country, *n* (%)**				
US	28 (40.6)	4 (12.1)	25 (80.6)	100 (43.1)
Outside US	22 (31.9)	19 (57.6)	6 (19.4)	129 (55.6)
Not Specified (Country)	19 (27.5)	10 (30.3)	0 (0.0)	3 (1.3)

Abbreviation: US, United States.

### 3.2 Immune‐Related Adverse Events

The distribution of immune‐related adverse events (irAEs) is summarized in Table 2. The most frequently reported category was skin and subcutaneous tissue disorders, accounting for 118 cases (32.3%), followed by endocrine disorders, with 66 cases (18.1%). Across all types of events, pembrolizumab was associated with the highest number of irAE reports. Among individual adverse reactions, rash was the most reported (*n* = 50, 13.7%), followed by pruritus (*n* = 34, 9.3%). Most other reactions had fewer than ten reports, with the exception of colitis associated with pembrolizumab, which was reported in 14 cases.

**TABLE 2:** Immune‐related adverse events.

Reaction	Atezolizumab	Avelumab	Nivolumab	Pembrolizumab
Skin and subcutaneous tissue disorders	27 (7.4)	7 (1.9)	6 (1.6)	78 (21.4)
Endocrine disorders	13 (3.6)	6 (1.6)	5 (1.4)	42 (11.5)
Gastrointestinal disorders	16 (4.4)	6 (1.6)	6 (1.6)	41 (11.2)
Renal and urinary disorders	9 (2.5)	3 (0.8)	4 (1.1)	14 (3.8)
Nervous system disorders	6 (1.6)	1 (0.3)	3 (0.8)	25 (6.8)
Cardiac disorders	4 (1.1)	5 (1.4)	1 (0.3)	17 (4.7)
Respiratory and thoracic mediastinal disorders	3 (0.8)	1 (0.3)	3 (0.8)	11 (3.0)
Musculoskeletal and connective tissue disorders	1 (0.3)	2 (0.5)	4 (1.1)	13 (3.6)
Eye disorders	0 (0.0)	0 (0.0)	0 (0.0)	2 (0.5)
Other	12 (3.3)	10 (2.7)	5 (1.4)	64 (17.5)
Rash	10 (2.7)	3 (0.8)	3 (0.8)	34 (9.3)
Pruritus	12 (3.3)	3 (0.8)	2 (0.5)	17 (4.6)
Hypothyroidism	1 (0.3)	4 (1.1)	3 (0.8)	7 (1.9)
Hyperthyroidism	6 (1.6)	1 (0.3)	1 (0.3)	3 (0.8)
Colitis	3 (0.8)	1 (0.3)	3 (0.8)	14 (3.8)
Hepatitis	4 (1.1)	1 (0.3)	1 (0.3)	2 (0.5)
Nephritis	5 (1.4)	1 (0.3)	0 (0.0)	3 (0.8)
Myocarditis	3 (0.8)	2 (0.5)	1 (0.3)	9 (2.5)
Pneumonitis	3 (0.8)	1 (0.3)	3 (0.8)	9 (2.5)
Myositis	2 (0.5)	3 (0.8)	0 (0.0)	4 (1.1)

*Note:* Percentages are calculated within each drug‐specific FAERS report group.

**FIGURE 2:**
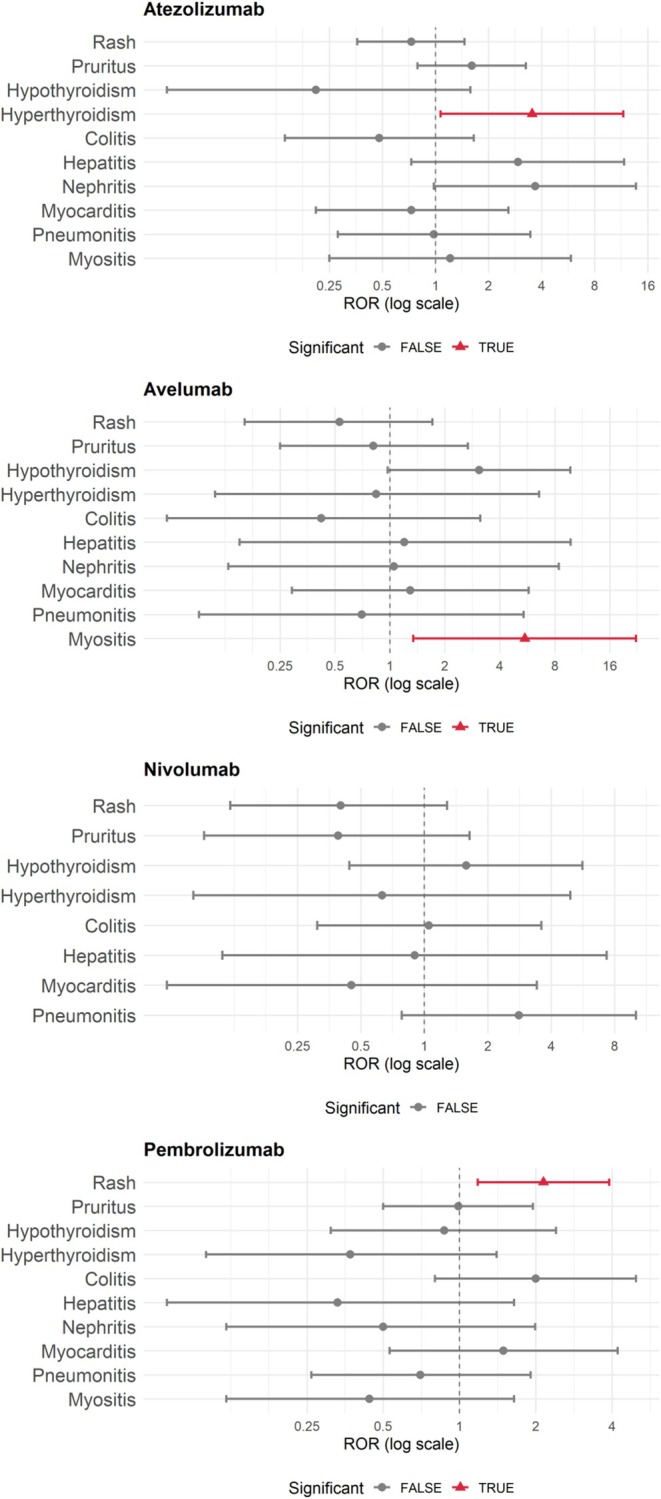
Forest plots of ROR by Drug and Adverse Reaction.

Results of the disproportionality analysis are presented in Table 3. Based on established criteria for signal detection, statistically significant safety signals were identified for hyperthyroidism with atezolizumab (PRR: 3.51 [95% CI: 1.07–11.45]; ROR: 3.53 [95% CI: 1.07–11.61]; IC: 1.10 [95% CI: 0.33–1.87]), rash with pembrolizumab (PRR: 2.11 [95% CI: 1.17–3.80]; ROR: 2.14 [95% CI: 1.18–3.90]; IC: 0.44 [95% CI: 0.17–0.71]), and myositis with avelumab (PRR: 5.30 [95% CI: 1.35–20.82]; ROR: 5.47 [95% CI: 1.34–22.29]; IC: 1.95 [95% CI: 0.65–3.25]). Forest plots depicting the RORs for each reaction are shown in Figure 2.

**TABLE 3:** Disproportionality analysis.

Reaction	Atezolizumab			Avelumab			Nivolumab			Pembrolizumab		
	PRR (95% CI)	ROR (95% CI)	IC (95% CI)	PRR (95% CI)	ROR (95% CI)	IC (95% CI)	PRR (95% CI)	ROR (95% CI)	IC (95% CI)	PRR (95% CI)	ROR (95% CI)	IC (95% CI)
Rash	0.73 (0.37, 1.45)	0.73 (0.36, 1.46)	−0.35 (−1.14, 0.44)	0.54 (0.17, 1.71)	0.53 (0.16, 1.71)	−0.83 (−2.4, 0.75)	0.4 (0.13, 1.29)	0.4 (0.12, 1.28)	−1.19 (−2.77, 0.39)	**2.11 (1.17, 3.8)**	**2.14 (1.18, 3.9)**	**0.44 (0.17, 0.71)**
Pruritus	1.59 (0.79, 3.2)	1.61 (0.79, 3.26)	0.47 (−0.18, 1.12)	0.81 (0.25, 2.64)	0.81 (0.25, 2.67)	−0.27 (−1.82, 1.28)	0.39 (0.09, 1.64)	0.39 (0.09, 1.64)	−1.22 (−3.15, 0.72)	0.99 (0.51, 1.94)	0.99 (0.5, 1.95)	0 (−0.49, 0.48)
Hypothyroidism	0.21 (0.03, 1.58)	0.21 (0.03, 1.58)	−1.93 (−4.67, 0.8)	3.05 (0.98, 9.51)	3.08 (0.97, 9.75)	**1.32 (0.12, 2.52)**	1.58 (0.45, 5.56)	1.58 (0.44, 5.63)	0.55 (−0.91, 2)	0.87 (0.32, 2.39)	0.87 (0.31, 2.4)	−0.1 (−0.88, 0.67)
Hyperthyroidism	**3.51 (1.07, 11.45)**	**3.53 (1.07, 11.61)**	**1.1 (0.33, 1.87)**	0.84 (0.11, 6.52)	0.84 (0.11, 6.57)	−0.23 (−2.92, 2.46)	0.63 (0.08, 4.91)	0.63 (0.08, 4.93)	−0.59 (−3.28, 2.1)	0.37 (0.1, 1.4)	0.37 (0.1, 1.4)	−0.88 (−2.27, 0.51)
Colitis	0.49 (0.14, 1.65)	0.48 (0.14, 1.65)	−0.84 (−2.34, 0.67)	0.42 (0.06, 3.11)	0.42 (0.06, 3.12)	−1.16 (−3.92, 1.59)	1.05 (0.31, 3.55)	1.05 (0.31, 3.59)	0.06 (−1.44, 1.57)	1.99 (0.8, 4.91)	2 (0.8, 4.97)	0.41 (−0.02, 0.84)
Hepatitis	2.92 (0.73, 11.65)	2.94 (0.73, 11.77)	0.97 (−0.02, 1.97)	1.2 (0.15, 9.7)	1.2 (0.15, 9.78)	0.23 (−2.41, 2.87)	0.9 (0.11, 7.3)	0.9 (0.11, 7.34)	−0.13 (−2.77, 2.51)	0.33 (0.07, 1.64)	0.33 (0.07, 1.64)	−1 (−2.74, 0.73)
Nephritis	3.65 (0.98, 13.56)	3.68 (0.98, 13.73)	**1.12 (0.29, 1.96)**	1.05 (0.13, 8.35)	1.05 (0.13, 8.42)	0.06 (−2.6, 2.72)	NA	NA	NA	0.5 (0.12, 1.98)	0.5 (0.12, 1.99)	−0.59 (−1.92, 0.74)
Myocarditis	0.73 (0.21, 2.58)	0.73 (0.21, 2.59)	−0.35 (−1.81, 1.11)	1.29 (0.29, 5.68)	1.29 (0.29, 5.76)	0.32 (−1.53, 2.18)	0.45 (0.06, 3.41)	0.45 (0.06, 3.42)	−1.04 (−3.77, 1.69)	1.49 (0.53, 4.17)	1.49 (0.53, 4.21)	0.26 (−0.34, 0.85)
Pneumonitis	0.98 (0.28, 3.39)	0.98 (0.28, 3.46)	−0.03 (−1.49, 1.43)	0.71 (0.09, 5.29)	0.7 (0.09, 5.4)	−0.46 (−3.18, 2.26)	2.74 (0.8, 9.39)	2.81 (0.78, 10.1)	1.27 (−0.17, 2.71)	0.71 (0.27, 1.88)	0.7 (0.26, 1.91)	−0.2 (−0.82, 0.42)
Myositis	1.21 (0.25, 5.77)	1.21 (0.25, 5.88)	0.22 (−1.54, 1.97)	**5.3 (1.35, 20.82)**	**5.47 (1.34, 22.29)**	**1.95 (0.65, 3.25)**	NA	NA	NA	0.44 (0.12, 1.63)	0.44 (0.12, 1.64)	−0.54 (−1.59, 0.51)

*Note:* Bold values indicate statistically significant disproportionality, defined as 95% confidence intervals not including 1 for PRR and ROR, and not including 0 for IC.

Abbreviations: 95% CI, 95% confidence interval; IC, information component; PRR, proportional reporting ratio; ROR, reporting odds ratio.

While not reaching the threshold for statistical significance, nephritis with atezolizumab approached the signal criteria (PRR: 3.65 [95% CI: 0.98–13.56]; ROR: 3.68 [95% CI: 0.98–13.73]; IC: 1.12 [95% CI: 0.29–1.96]). A similar borderline pattern was observed for hypothyroidism with avelumab (PRR: 3.05 [95% CI: 0.98–9.51]; ROR: 3.08 [95% CI: 0.97–9.75]; IC: 1.32 [95% CI: 0.12–2.52]). No other irAEs met the criteria for a statistically significant safety signal.

## 4 Discussion

This study evaluated immune‐related adverse events associated with four immune checkpoint inhibitors approved for the treatment of bladder cancer, using data from the FAERS database. Consistent with findings from prior clinical trials, dermatologic and endocrine toxicities were the most frequently reported irAEs [15, 19, 22, 23]. For example, up to 40% of patients in clinical trials have been reported to experience dermatological adverse events [24].

In our analysis, rash emerged as a statistically significant safety signal associated with pembrolizumab monotherapy (PRR: 2.11 [95% CI: 1.17–3.80]; ROR: 2.14 [95% CI: 1.18–3.90]; IC: 0.44 [95% CI: 0.17–0.71]). This suggests that rash may be more frequently associated with pembrolizumab than with other ICIs when used alone. Several clinical trials have documented rash as a common adverse effect of pembrolizumab. Two trials, for instance, reported incidence rates of 13.2% (40/302) and 10.9% (29/266) in the pembrolizumab arm, compared with 7.0% (24/342) and 6.3% (16/255), respectively, in the chemotherapy arm [17, 23]. A separate study comparing pembrolizumab to placebo reported rash in 21.8% vs. 7.7% of patients, respectively [25]. Moreover, a prior real‐world study of FAERS reports also identified rash as a frequently reported event among patients receiving pembrolizumab [26]. Rash associated with ICI therapy can present in various forms, including maculopapular and psoriasiform rash, which are the most commonly described in the literature [27]. Similarly, in our analysis, multiple rash subtypes were reported using various MedDRA Preferred Terms, such as rash erythematous and rash maculopapular. Detailed classifications of these terms are provided in Table S1.

Rashes associated with ICI therapy typically appear after a few treatment cycles and are generally nonspecific and of low‐grade severity [28]. These dermatologic toxicities most commonly affect the trunk and the extremities [27, 28]. Management of rash depends on its severity, which is classified according to the National Cancer Institute's Common Terminology Criteria for Adverse Events. Under this system, rash is categorized as grade 1 if it involves less than 10% of body surface area (BSA), grade 2 for 10%–30% BSA, and grade 3 for greater than 30% BSA or when there is a marked impact on quality of life [28]. Mild (grade 1–2) rashes are typically managed with topical moisturizers and high‐potency topical corticosteroids, while more severe cases (grade 3) may require systemic corticosteroids and temporary discontinuation of ICI therapy. For life‐threatening or grade 4 reactions, permanent discontinuation of immunotherapy is often necessary [27, 28]. However, in most cases, rash does not necessitate permanent treatment discontinuation [24]. Nonetheless, even lower‐grade rashes may significantly impair a patient's quality of life and warrant proactive management [29].

The significant association of rash with pembrolizumab observed in our analysis may be partially explained by the higher absolute number of adverse event reports for pembrolizumab. Although pembrolizumab was not the first ICI approved for bladder cancer, its approval came only one year after that of atezolizumab [30, 31]. Moreover, pembrolizumab is recommended across multiple indications and disease stages in bladder cancer, which, combined with its longer presence on the market (over eight years), may have contributed to its higher reporting volume [6]. Differences in reporting practices and global usage patterns may also explain the disproportionate number of reports associated with pembrolizumab. Therefore, this signal should be interpreted with caution, as it may reflect differences in reporting volume and patterns rather than a true difference in risk.

In addition to dermatologic toxicities, endocrine disorders, particularly hypothyroidism and hyperthyroidism, were also frequently reported in our analysis. Notably, hyperthyroidism was identified as a significant adverse event associated with atezolizumab (PRR: 3.51 95% CI: 1.07–11.45; ROR: 3.53 [95% CI: 1.07–11.61]; IC: 1.1 [95% CI: 0.33–1.87]). Clinical data suggest that endocrine irAEs occur in up to 40% of patients receiving ICIs [32]. Specifically, hyperthyroidism has been reported more frequently in patients treated with atezolizumab, with observed incidences of 4.8% (17/354) and 4.6% (17/390) compared to 1.8% (7/390) and 0% (0/397) among patients receiving chemotherapy and observation, respectively. Interestingly, meta‐analytic data indicate that the incidence of hyperthyroidism with PD‐L1 inhibitors (e.g., atezolizumab) is lower than with PD‐1 inhibitors, raising questions about our observed signal [33]. Nonetheless, our findings are consistent with a previous FAERS‐based analysis that reported a risk ratio of 6.4 for hyperthyroidism with atezolizumab compared to other drugs [34]. This discrepancy between clinical trial and post‐marketing data may reflect differences in patient selection, duration of exposure, geographic variability, or reporting practices. Additionally, biological differences in immune activation between PD‐1 and PD‐L1 blockades may influence thyroid autoimmunity in ways not fully captured in controlled trials. These findings highlight the importance of real‐world pharmacovigilance data in complementing clinical trial evidence and warrant further mechanistic investigation.

The American Society of Clinical Oncology (ASCO) has issued specific guidelines for the diagnosis and management of hyperthyroidism induced by ICI therapy. Routine monitoring of thyroid‐stimulating hormone (TSH) and free thyroxine (FT4) every 4 to 6 weeks is recommended during treatment. For grade 1 toxicities characterized by laboratory abnormalities without significant symptoms, continued monitoring of TSH levels is advised, and ICI therapy can typically be continued without interruption. In grade 2 cases, which involve moderate symptoms, temporary withholding of ICI therapy is recommended along with symptomatic management using beta‐blockers, hydration, and supportive care. For grade 3 or 4 reactions, which present with severe or life‐threatening symptoms, similar treatment strategies are employed, although hospitalization may be necessary for close monitoring and supportive interventions [35].

In our analysis, a statistically significant signal was also observed for myositis in association with avelumab. Among the seven completed clinical trials of avelumab in bladder cancer registered on ClinicalTrials.gov, only one study evaluated avelumab monotherapy, and this trial reported an identical incidence of myositis (0.6%) in both the avelumab and best supportive care arms [19]. A case report has previously documented avelumab‐associated myositis, providing some clinical support for this observed signal [36]. Overall, however, the incidence of ICI‐associated myositis remains low, with pooled estimates suggesting a rate of approximately 0.10%. Management of myositis depends on severity: Grade 1–2 toxicities are generally treated with oral corticosteroids, whereas grade 3–4 reactions may necessitate intravenous immunosuppressive therapy [37]. The identification of a safety signal for myositis in our real‐world analysis emphasizes the value of post‐marketing surveillance studies in capturing rare adverse events that may be underreported or missed in clinical trial populations.

Our study also identified a possible association between hypothyroidism and avelumab and between nephritis with atezolizumab; however, these irAEs did not meet the predefined criteria to qualify as statistically significant safety signals. This may be attributable to a limited number of reports, relatively shorter time on the market for certain agents, or variability in spontaneous reporting practices. As the volume of post‐marketing data in the FAERS database continues to grow, the risk estimates for these events may change, underscoring the need for continued pharmacovigilance and future research to better characterize these associations. These signals should not be depended upon in a clinical context and are intended solely to generate hypotheses for future studies.

In our dataset, a greater number of adverse event reports involved male patients, which is consistent with the known sex distribution of bladder cancer. Furthermore, the majority of the reports were submitted by healthcare professionals, lending credibility to the accuracy and seriousness of the reported events. While previous studies have documented the occurrence of irAEs with ICIs, our analysis contributes to the existing literature by offering a comparative assessment of four ICI agents in a real‐world setting. These findings suggest that specific organ systems may be differentially affected by different ICI therapies. Although product labeling for ICIs includes warnings about the potential for irAEs, these data highlight the need for clinician awareness and proactive patient counseling regarding the spectrum of adverse events and available management strategies. However, these findings should not be relied upon for clinical decision‐making due to the limitations of the FAERS database and should instead be considered as hypothesis‐generating safety signals.

There are several limitations to this study. First, the FAERS database is a spontaneous reporting system and is therefore subject to reporting bias, underreporting, and duplicate submissions, which can lead to either overestimation or underestimation of the true risk of adverse events. Second, FAERS lacks detailed information on factors that can significantly influence individual susceptibility to immune‐related adverse events, such as patient demographics, drug dosage, treatment duration, and timing of administration. Third, the four ICIs evaluated in this study were approved at different time points, resulting in varying durations of market exposure, which may affect the volume and distribution of reports submitted for each agent and consequently the strength of the safety signals detected. Lastly, while disproportionality analysis using FAERS can identify potential safety signals, it cannot establish causal relationships and does not constitute a direct comparative safety assessment, as the database does not include data on drug exposure rates or patient denominators necessary for calculating incidence.

## 5 Conclusion

In conclusion, this disproportionality analysis identified a safety signal for rash with pembrolizumab, as well as signals for hyperthyroidism with atezolizumab and myositis with avelumab. It is important to note that the signal for pembrolizumab may be influenced by the longer time on the market and the higher frequency of use. These findings contribute valuable real‐world evidence regarding potential differences in immune‐related adverse events based on the specific type of ICI agent used. The study underscores the critical role of post‐marketing surveillance systems such as FAERS in detecting safety signals that may not be evident in clinical trials. Clinicians should remain vigilant about the risk of irAEs during ICI therapy in bladder cancer and engage in proactive patient counseling to mitigate adverse outcomes and preserve quality of life. Future research should prioritize comparative pharmacoepidemiologic studies that incorporate real‐world drug exposure data and control for confounding variables to more accurately quantify and compare the safety profiles of individual ICIs.

## Author Contributions

**Lorenzo Villa Zapata:** methodology, supervision, writing – review and editing. **Pooja Gokhale:** conceptualization, data curation, formal analysis, writing – original draft, methodology.

## Funding

This study was supported by no funding was received to assist with the preparation of this manuscript.

## Disclosure

The authors have nothing to report.

## Conflicts of Interest

The authors declare no conflicts of interest.

## Supporting information


**Table S1:** Preferred terms.

## Data Availability

The data that support the findings of this study are openly available in FAERS Dashboard at https://www.fda.gov/drugs/fdas‐adverse‐event‐reporting‐system‐faers/fda‐adverse‐event‐reporting‐system‐faers‐public‐dashboard.
