# Supplementary material for: Immune‐Related Adverse Events in Bladder Cancer Patients Treated With Immune Checkpoint Inhibitors: Insights From a FAERS Disproportionality Analysis
Source: Cancer Med. 2026 Apr 2;15(4):e71777. doi: 10.1002/cam4.71777 (PMC13045409; doi:10.1002/cam4.71777)
Supplement: Supplementary file 1 — Table S1: Preferred terms. [file CAM4-15-e71777-s001.docx]

Immune-Related Adverse Events in Bladder Cancer Patients Treated with Immune Checkpoint Inhibitors: Insights from a FAERS Disproportionality Analysis

Corresponding author:

Lorenzo Villa Zapata: [Lorenzo.VillaZapata@uga.edu](mailto:Lorenzo.VillaZapata@uga.edu)

Department of Clinical and Administrative Pharmacy, University of Georgia College of Pharmacy, Athens, GA, USA

Supplementary Table S1: Preferred terms

| Disorders | Terms reported in FAERS | Preferred Terms (PTs) |
| --- | --- | --- |
| Skin and subcutaneous tissue disorders | Rash | Rash |
|  | Rash Erythematous |  |
|  | Rash Papular |  |
|  | Rash Pruritic |  |
|  | Rash Maculo-Papular |  |
|  | Genital Rash |  |
|  | Pruritus | Pruritus |
|  | Senile Pruritus |  |
|  | Ear Pruritus |  |
|  | Dermatitis Acneiform | Dermatitis |
|  | Dermatitis Bullous |  |
|  | Seborrhoeic Dermatitis |  |
|  | Immune-Mediated Dermatitis |  |
|  | Erythema | Erythema |
|  | Erythema Multiforme |  |
|  | Psoriasis | Psoriasis |
|  | Guttate psoriasis |  |
|  | Urticaria | Urticaria |
|  |  |  |
| Gastrointestinal disorders | Immune-Mediated Enterocolitis | Colitis |
|  | Colitis |  |
|  | Enterocolitis |  |
|  | Colitis Microscopic |  |
|  | Colitis Ulcerative |  |
|  | Eosinophilic Colitis |  |
|  | Pseudomembranous Colitis |  |
|  | Colitis Ischaemic |  |
|  | Hepatitis | Hepatitis |
|  | Immune-Mediated Hepatitis |  |
|  | Immune-Mediated Hepatic Disorder |  |
|  | Cholestasis | Cholestasis |
|  | Stomatitis | Stomatitis |
|  | Pancreatitis | Pancreatitis |
|  | Immune-Mediated Cholangitis | Cholangitis |
|  | Immune-Mediated Gastritis | Gastritis |
|  |  |  |
| Nervous system disorders | Encephalitis | Encephalitis |
|  | Autoimmune Encephalitis |  |
|  | Myelitis | Myelitis |
|  | Optic Neuritis | Optic Neuritis |
|  |  |  |
| Endocrine disorders | Hyperthyroidism | Hyperthyroidism |
|  | Hypothyroidism | Hypothyroidism |
|  | Autoimmune Hypothyroidism |  |
|  | Adrenal Insufficiency | Adrenal Insufficiency |
|  | Immune-Mediated Adrenal Insufficiency |  |
|  | Hypophysitis | Hypophysitis |
|  | Immune-Mediated Hypophysitis |  |
|  | Thyroiditis | Thyroiditis |
|  | Autoimmune Thyroiditis |  |
|  | Hypogonadism | Hypogonadism |
|  | Hypoparathyroidism | Hypoparathyroidism |
|  | Graves' disease | Graves' disease |
|  |  |  |
| Cardiac disorders | Myocarditis | Myocarditis |
|  | Immune-Mediated Myocarditis |  |
|  |  |  |
| Respiratory and thoracic mediastinal disorders | Pneumonitis | Pneumonitis |
|  | Autoimmune Lung Disease | Autoimmune Lung Disease |
|  | Immune-Mediated Lung Disease | Immune-Mediated Lung Disease |
|  |  |  |
| Renal and urinary disorders | Nephritis | Nephritis |
|  | Autoimmune Nephritis |  |
|  | Tubulointerstitial Nephritis |  |
|  | Pyelonephritis | Pyelonephritis |
|  | Immune-Mediated Renal Disorder | Immune-Mediated Renal Disorder |
|  | Cystitis | Cystitis |
|  | Immune-Mediated Cystitis |  |
|  |  |  |
| Muscoluskeletal and connective tissue disorders | Autoimmune Myositis | Myositis |
|  | Immune-Mediated Myositis |  |
|  | Dermatomyositis |  |
|  | Paraneoplastic Dermatomyositis |  |
|  | Myasthenia Gravis | Myasthenia Gravis |
|  | Myasthenia Gravis Crisis |  |
|  | Arthritis Reactive | Arthritis |
|  | Immune-Mediated Arthritis |  |
|  |  |  |
| Eye disorders | Keratitis | Keratitis |
|  | Uveitis | Uveitis |
|  | Conjunctivitis | Conjunctivitis |
|  |  |  |
| Others | Sarcoidosis | Sarcoidosis |
|  | Vasculitis | Vasculitis |
|  | Toxic Epidermal Necrolysis | Toxic Epidermal Necrolysis |
|  | Sjogren's Syndrome | Sjogren's Syndrome |
|  | Stevens-Johnson Syndrome | Stevens-Johnson Syndrome |
|  | Immune-Mediated Adverse Reaction | Immune-Mediated Adverse Reaction |
|  | Immune System Disorder | Immune System Disorder |
|  | Autoimmune Haemolytic Anaemia | Autoimmune Haemolytic Anaemia |
